# Supplementary material for: Patterns of Intron Gain and Loss in Fungi
Source: PLoS Biol. 2004 Nov 30;2(12):e422. doi: 10.1371/journal.pbio.0020422 (PMC532390; doi:10.1371/journal.pbio.0020422)
Supplement: Table S1 — Also available at http://genes.mit.edu/NielsenEtAl/. (4.3 MB ZIP). [file pbio.0020422.st001.zip › NielsenEtAl/html/552.html]

AN2518.1.NCU04252.1.MG09552.1.FG10345.1


```
 CLUSTAL W (1.82) Multiple Sequence Alignments - Introns Inserted


Sequence 1: MG09552.1	715 aa
Sequence 2: FG10345.1	705 aa
Sequence 3: NCU04252.1	675 aa
Sequence 4: AN2518.1	527 aa
Alignment Length: 721 aa
Number Identitical Residues: 273 aa
Alignment Score (without introns) 15466


MG09552.1 	MAAR-GNSLRDRQIA1SLRKIFNLNNEVEASDNEESLASGALNPSSPILNADGEPIWKVL
NCU04252.1	MAPTPGNSLRDKQIL1SIKKLLNLNQPLEPTDGDD--SNGFQSASAPLLKD--IPTWKIL
FG10345.1 	MATK-GNSLRDRQIA1SLKKILNLNETVESSEADEAHANGLLAPVAPILDADGNPIWKVL
AN2518.1  	------MSLRDRQVA1SIQKILNLNHDPRPAEDATHEASAQGLISTPILNEDGDPIWKIL
          	       ****:*:  *::*::***.  ..::     :..     :*:*. .. * **:*

MG09552.1 	VFDDLGRDVISSVLRVSDLRSMGVTMHM2HISASRHPIPDVPVMYLLEPHQKNLEAITND
NCU04252.1	VFDELGRNVISPVLQVSDLRSMGVTLHV~SIAANRQPIPDVDAIYLIEPNAKNLQQITND
FG10345.1 	VFDDLGRDVISSVMRVSDLRAMGVTMHM2HIGGARHPIPDVPVIYLLEPNAQNLQAITSD
AN2518.1  	VFDNLGRDVISSVLRVNDLRAWGVTIHL2------YPIPDVPVVYLVEPTASNIQLIAND
          	***:***:***.*::*.***: ***:*:        ***** .:**:**  .*:: *:.*

MG09552.1 	LQKGLYSPAYINFLSSIPRPLLEDFATQTAAAGTSDHIAQLFDQYLNFVVAEPDLFSLGM
NCU04252.1	LQKNLYNSASINFLSSVPRPLLEDFAAQTAVAGTSEKIAQIFDQYLNFIVPEPDLFSLGM
FG10345.1 	LQKGLYTPAYINFLSSLPRVLLEDFATQTATAGTSEHIAQLFDQYLNFIVAEPDLFSLGM
AN2518.1  	LQRGLYSPAYVNFLSSIPRPLLEDFASQIATTGTAEHIAQVYDQYLNFIVAEPDLFSLGL
          	**:.**..* :*****:** ******:* *.:**:::***::******:*.********:

MG09552.1 	QKEHTYWALNSAKTNDEELDNVVDRIVSGLFSVIVTMG1SIPIIRCPKSAAAEMISAKLD
NCU04252.1	QKEHTYWALNSAKTQDEELDRVIDRIVSGLFSVVVTLG~VIPIIRCPRGAAAEMVATKLD
FG10345.1 	QKEHTYWALNSAATSDEELDRVVDKIVSGLFSVIATMG1VIPIIRCPKGAAAEMVAAQLD
AN2518.1  	G-NDAYWRINSAKTSDADLDEIVDKIVSGLFSVSVTMG1AIPIIRCPKGGAAELIATKLD
          	  :.:** :*** *.* :**.::*:******** .*:*  *******:..***:::::**

MG09552.1 	RKLRDHILNSKDNLFSASNRSGSSSATPGPRPVLIILDRNVDLTPMLSHSWTYQSLVHDV
NCU04252.1	RKLRDHILNSKDNLFSAHRAAASSTGTP--KPLLVIMDRNIDLIPMLSHSWTYQSLCADV
FG10345.1 	RKLRDHILNSKDNLFSGP-RPNASSGTHSSRPVLILLDRNIDLIPMLSHSWTYQSLVHDV
AN2518.1  	RKLRDHILNSKDNLFSAN--KKSTPGVPSSRPVLIIVDRNVDLVPMLSHSWTYQSLVQDV
          	****************.     ::... ..:*:*:::***:** ************  **

MG09552.1 	LNMK-LNRITIETPVDESNPAKGVTRKAYDLNANDFFWERNAGAPFPQ~VAEDIDAELTK
NCU04252.1	FGLSESNRITIESPVDSNNPAKGTNKKTYDLAADDFFWAKNSCLPFPQ0VAEDIDIELTK
FG10345.1 	LNMK-LNRITIETPVEEGNPEKGPTKKGYDLTTSDFFWAKNAGSPFPQ~VAEDIDAELTK
AN2518.1  	LQMR-LNRITVETPVDESNPAAGTTKKAYDLGSNDFFWKRNAGAPFPH~VAEDIDAELTR
          	: :   ****:*:**:..**  * .:* *** :.**** :*:  ***: ****** ***:

MG09552.1 	YKEEAAAITKKTGVTDLEDLQNDTSASAQHLKAAITLLPELRERKATLDMHMNILAALLT
NCU04252.1	YKEEADALTKKTGVRDLDHFEQDSSASAQHLKAAVTLLPELRARKATLDMHMNILAAILG
FG10345.1 	YKEDTAAITKKTGVTDLEDLQADTSASAQHLKAAITLLPEMRERKGILDMHMNILAALLT
AN2518.1  	YKEDATEITRKTGASSIEDLQADTSASAQHLKAAITLLPELRERKSILDMHMNIATALLK
          	***::  :*:***. .::.:: *:**********:*****:* **. ******* :*:* 

MG09552.1 	GIKNRQLDNYFQIEETVTKQTKVQILETIKDDNKGNNPVDKLRLFIIWFLSAEQDLSRQE
NCU04252.1	EIQSRQLDNYFQLEENVFKQTKAQVLDQIKTADKGK-PEDYLRLFVIWYLSTEQDVSRQE
FG10345.1 	GIKDRQLDNYFQLEENVVKQTKAQIMEIIKDDNKGTDPVDKLRLFIIWFLSTEQEVNRQE
AN2518.1  	GIKDRQLDNFFELEENITKQSKAQILELISDPTKGTNPTDKLRIFLIWFLSTETELSRAD
          	 *:.*****:*::**.: **:*.*::: *.   **..* * **:*:**:**:* ::.* :

MG09552.1 	WQQFEEALVATG-VDVTCLPYIRQ2VRATTKMTQLTTINSSSSQPQQ---SSDLFGRFSN
NCU04252.1	WTQFEEALAAKG-CDTTCLSYIKQ2VRATTKMTQLTTVNNPSSSNQQQSGSSDLFNRFSA
FG10345.1 	FESFEKALSEAG-ADVSSLPYVRQ2VRATTKMTQLTTINN-TAQPAQ---TSDLFGRFSS
AN2518.1  	ISQFEEALTRTGVQDISPIAYVKQ~VRELTRMTMMTTATA--APQQQ---SSDLFRGFSS
          	  .**:**   *  * : :.*::* **  *:** :** .   :   *   :****  ** 

MG09552.1 	IGSNIGSRLRDQLKESGVPTNAISANFDSIIGGIKTFLPVNRDLTVTKIVESIMDPSAAS
NCU04252.1	ISS----RLTDRLKETGVPTGALSNNVASLLGGIKNFLPEDRDLTVTKITESLMDPSAAS
FG10345.1 	MSS----RLTDRLKESGVPT-GLSSNFESLISGVKNFLPADRDFTVTKIVESIMDPSSAS
AN2518.1  	LSN----RLTDRITSAP-------------------------------------------
          	:..    ** *::..:                                            

MG09552.1 	SSAIAKTEHYLYFDPRSANARGTVPPP~SAVRSGTVPTSTPGGLPGAGGPGAAASFGQRR
NCU04252.1	SSAIAKTEHYLYFDPRSANARGTMPQP2--------------------------------
FG10345.1 	SSAIAKTEHFLYYDPRSANARGTMPPP~SAVRSGAG-TSTPGGMPGSQVPGQTASFGQRR
AN2518.1  	---------------------------~--------------------------------
          	                                                            

MG09552.1 	QGFTEAVVFTVGGGSMDEYGNLQEWVARTSGSGDRVRRRVVYGSTEMVNATEFISQELDR
NCU04252.1	QGFSEAIVFMVGGASTAEYSNLQEWAARTTTG-DRAKRRVIYGGTELLNAGSFIKEELER
FG10345.1 	QGFSEAVVFTVGGGSMDEYGNLQEWVSRTGG--DRAKKRVVYGSTEMVNAAEFITEELDT
AN2518.1  	------------------------------------------------------------
          	                                                            

MG09552.1 	LGKEVAS
NCU04252.1	LGKEVS-
FG10345.1 	LGKEVAS
AN2518.1  	-------
          	
```
